# Complete Pathological Response to Neoadjuvant Pembrolizumab in a Patient With Chemoresistant Upper Urinary Tract Urothelial Carcinoma: A Case Report

**DOI:** 10.3389/fonc.2020.564714

**Published:** 2020-09-24

**Authors:** Daiki Ikarashi, Shigehisa Kitano, Kazuyuki Ishida, Tetsuya Nakatsura, Hitoshi Shimodate, Takashi Tsuyukubo, Daichi Tamura, Renpei Kato, Tamotsu Sugai, Wataru Obara

**Affiliations:** ^1^Department of Urology, Iwate Medical University School of Medicine, Iwate, Japan; ^2^Division of Cancer Immunotherapy Development, Advanced Medical Development Center, The Cancer Institute Hospital of Japanese Foundation for Cancer Research Ariake, Tokyo, Japan; ^3^Division of Cancer Immunotherapy, Exploratory Oncology Research & Clinical Trial Center, National Cancer Center, Chiba, Japan; ^4^Department of Pathology, Iwate Medical University School of Medicine, Iwate, Japan

**Keywords:** pembrolizumab, neoadjuvant, pathological complete response, ureteral carcinoma, chemoresistant

## Abstract

Treatment options as second-line therapy for advanced ureteral carcinoma are limited, and patients experiencing recurrence after first-line cisplatin-based chemotherapy have a poor prognosis. Recently, the programmed death-1 (PD-1) inhibitor pembrolizumab provided a better survival benefit with a complete response rate (9.2%) for chemoresistatant urothelial carcinoma. However, the dynamic changes of the cancer microenvironment about the cases of complete response are still unknown. We herein report a case of a 57-year-old man who had been diagnosed with localized, non-muscle-invasive bladder cancer (pT1N0M0, high grade), for which he underwent transurethral resection of the bladder cancer twice. Given that gemcitabine plus carboplatin as first-line neoadjuvant chemotherapy was unable to control left vesico-ureteral junction recurrence with muscle invasion (T3N0M0, high grade), the patient received the PD-1 inhibitor pembrolizumab as second-line neoadjuvant therapy in an attempt to stop tumor growth, which promoted dramatic tumor shrinkage without serious adverse effects and allowed subsequent nephroureterectomy and lymphadenectomy. To the best of our knowledge, this has been the first study to report that pembrolizumab administration before surgery for chemotherapy-resistant ureteral carcinoma promoted a pathological complete response, providing a better understanding of the cancer microenvironment after immunotherapy.

## Background

Although nephroureterectomy has been the standard treatment for upper urinary tract urothelial carcinoma (UTUC), surgery alone has been associated with a high risk for recurrence and 5-year survival rates of less than 50% (1). As such, a shift in the treatment paradigm to incorporate neoadjuvant chemotherapy among patients with high-risk UTUC has been advocated. A retrospective study by Porten et al. (2) in 2014 demonstrated that neoadjuvant cisplatin-based chemotherapy conferred improved overall and disease-specific survival. Despite the efficacy of first-line treatments for UTUC patients, a considerable number of patients have experienced disease progression during or after fist-line treatment, which would require second-line therapy. Although several second-line chemotherapeutic agents have been investigated for metastatic urothelial carcinoma, they have only presented marginal activity with an overall response rate of <20% and a median overall survival of <9 months with considerable toxicity profiles (3, 4). Recently, pembrolizumab, a humanized monoclonal immunoglobulin G (IgG)4 isotype antibody against the programmed death-1 (PD-1), had been approved as a second-line treatment for metastatic urothelial carcinoma after failure of platinum-based chemotherapy. Accordingly, one phase III trial, KEYNOTE-045 trial, showed that pembrolizumab provided better survival benefit, objective response rate (21.1%), and complete response rate (9.2%) than other chemotherapeutic agents, such as docetaxel, paclitaxel, and vinflunine (5, 6). Therefore, curative treatment has generally been targeted for advanced UTUC. However, few studies have utilized pembrolizumab as a presurgical treatment for chemoresistant advanced UTUC. Here, we report a case involving the neoadjuvant treatment of advanced UTUC with pembrolizumab and subsequent safe nephroureterectomy, resulting in complete pathological response as confirmed by immunohistochemistry.

## Case Presentation

**FIGURE 1:**
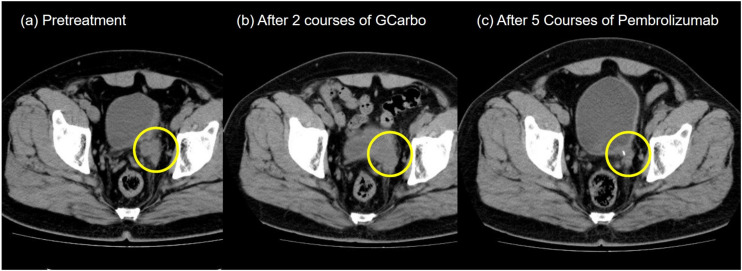
Pelvic computed tomography during treatment. **(a)** Pretreatment. A left ureteral tumor was observed. **(b)** After two courses of gemcitabine plus carboplatin chemotherapy. **(c)** After five courses of pembrolizumab.

**FIGURE 2:**
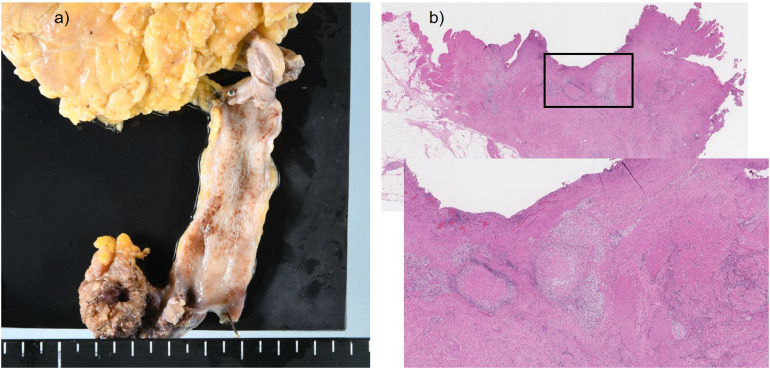
Surgical specimen and histopathological findings. **(a)** Only the scar tissue was shown at the lower ureteral site. **(b)** Accumulation of foamy histiocytes and infiltration of chronic inflammatory cells were observed around the necrotic tissue.

**FIGURE 3:**
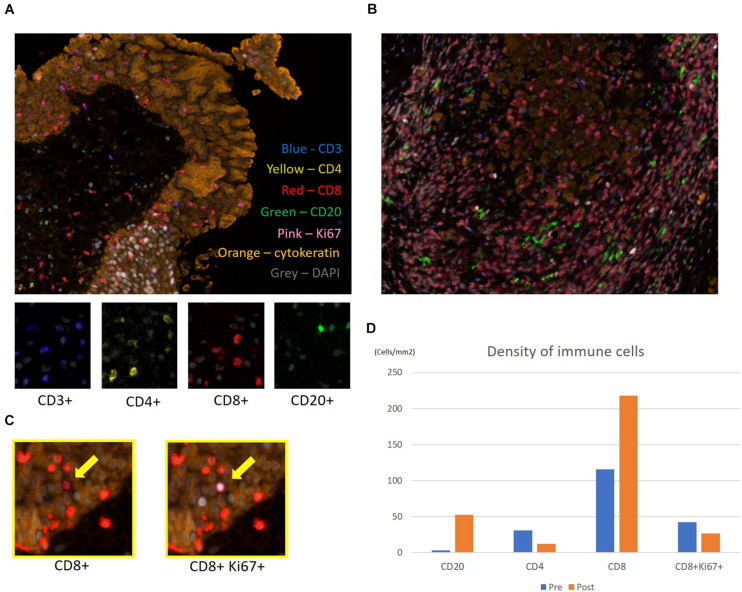
Multiplex fluorescence immunohistochemistry. Evaluation of immune cells before and after neoadjuvant therapy for upper urinary tract urothelial carcinoma. Representative multiplex fluorescence images of tumor-infiltrating T and B cells in the **(A)** biopsy specimen and **(B)** resected specimen. Nuclei, CD3, CD4, CD8, CD20, Ki67, and cytokeratin within the cells are shown in gray, blue, yellow, red, green, pink, and orange, respectively. **(C)** Ki67^*high*^ subpopulation of CD8 T cells (yellow arrows) was determined by visualizing nuclear Ki67 (pink) expression. **(D)** Evaluation of each immune cell’s density before and after pembrolizumab therapy in the biopsy and resected specimens.

**FIGURE 4:**
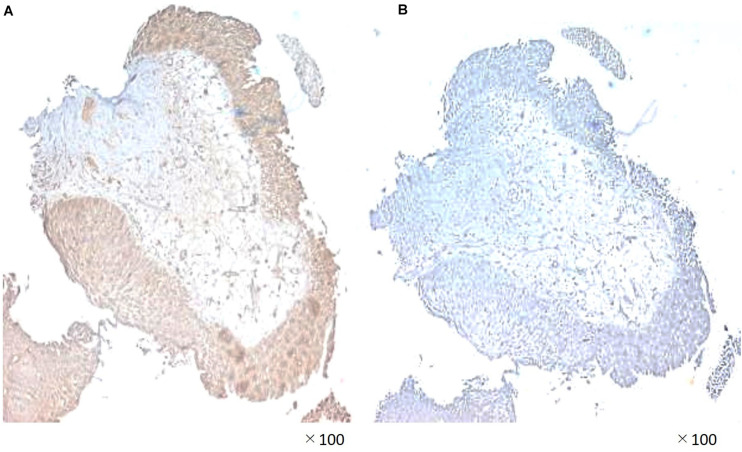
Immunohistochemical staining demonstrating **(A)** significant programmed death ligand-1 (PD-L1) expression and **(B)** lack of PD-L2 expession in the biopsy specimen.

A 55-year-old Japanese man who was a heavy smoker and had a history of two transurethral resections of bladder tumor (TURBT) for non-muscle-invasive bladder cancer presented to our hospital with macrohematuria. He had a medical history of hypertension, hyperuricemia, and chronic kidney disease. Accordingly, contrast-enhanced abdominal computed tomography examination 1.5 years after his last TURBT revealed a left lower ureteral tumor with hydronephrosis and atrophy of renal parenchyma (Figure 1a). Tumor biopsy results led to a diagnosis of high-grade, locally advanced urothelial carcinoma with a clinical stage of T3N0M0. Initially, gemcitabine plus carboplatin (GCarbo) as first-line neoadjuvant chemotherapy had been provided due to his impaired renal function (estimated glomerular filtration rate: 34.2 ml/min/1.73 m^2^). After two chemotherapy courses, the primary tumor and left external iliac lymph nodes became enlarged, while hematuria reappeared (Figure 1b). As such, pembrolizumab as second-line therapy was administered at a dose of 200 mg every 3 weeks. After five courses of pembrolizumab, CT showed 90% shrinkage of the tumor and left external iliac lymph nodes, which constituted a partial response (Figure 1c). Thereafter, the possibility of curative nephroureterectomy was discussed with the patient and with the multidisciplinary urological team. Given the significant shrinkage of the primary tumor and the absence of adverse events, left nephroureterectomy and lymph node dissection were performed. Histopathological findings showed expanded necrosis and infiltration of foamy histiocytes with no viable cells in the surgical specimens (Figure 2). Furthermore, evaluation of immunological changes before and after neoadjuvant therapy were performed using multiplex immunohistochemistry. Tumor-infiltrating CD8^+^ cells were sparsely found in the biopsy specimens but were remarkably increased in the resected specimens. However, the density of CD8^+^ cells with high Ki67 expression in the resected specimens were the same as that in biopsy specimens. On the other hand, despite tumor-infiltrating CD20^+^ B cells were not found in the biopsy specimen, they were observed surrounded by CD8^+^ cells in the resected specimens (Figure 3). To assess the microenvironment of the lesions, multiplexed fluorescent immunohistochemistry was conducted following the same method used in our previous study (7). Moreover, programmed death ligand-1 (PD-L1) and PD-L2 expressions in the biopsy specimens were also evaluated using immunohistochemistry. The expression of PD-L1 was positive for tumor cell; meanwhile, PD-L2 was negative for the tumor cell in biopsy specimens (Figure 4).

The perioperative course remained uneventful, and the patient was discharged 10 days after the surgery. No local recurrence or metastasis had been observed 1 year after stopping pembrolizumab therapy.

## Discussion

In May 2017, the US Food and Drug Administration had granted accelerated approval for pembrolizumab. The mechanism of action for pembrolizumab involves binding to the PD-1 receptor expressed on T lymphocytes and inhibiting its interaction with PD-L1 expressed on cancer cells. This subsequently inhibits immune evasion by cancer cells and maintains the antitumor effect of T lymphocytes. Currently, pembrolizumab, which provides fewer side effects compared to existing chemotherapies, has been widely used as a second-line therapy for advanced urothelial cancer after failure of first-line platinum-based chemotherapy. However, results of clinical trials have shown that not all cases benefit from pembrolizumab (5).

Although first-line neoadjuvant GCarbo showed no effect in the present patient, second-line pembrolizumab provided remarkable effects and subsequent safe nephroureterectomy. As a result of the clinical course, pembrolizumab played the role of neoadjuvant in this case. Indeed, Necchi et al. (8) reported that pembrolizumab was effective as a first-line neoadjuvant therapy for MIBC such that 21 (42%) of 46 patients achieved pT0 after only three cycles following radical cystectomy. Nonetheless, the efficacy of pembrolizumab as a neoadjuvant therapy for UTUC still remained unclear, while fewer than 10% of the patients enrolled in the KEYNOTE045 trial had UTUC (5).

Pembrolizumab enables to activate and enhance the cytotoxic T cells. This finding had been markedly observed in the resected ureter tumor, which showed tumor shrinkage during pembrolizumab treatment. Therefore, multiplex fluorescence immunohistochemical staining of biopsy specimen before chemotherapy and resected specimens following pembrolizumab was retrospectively conducted to evaluate immunological changes in the tumor microenvironment. Accordingly, tumor-infiltrating CD8^+^ cells were sparsely found in the biopsy specimen but were remarkably increased in the resected specimen following pembrolizumab. Tumeh et al. (9) reported that substantial infiltration of CD8^+^ cells into melanoma tissues was associated with a good response to pembrolizumab therapy. As such, infiltration of CD8^+^ cells in tumor may potentially predict the effectiveness of pembrolizumab in this case. Similar histological findings had been observed in renal cell carcinoma wherein nivolumab promoted a pathological complete remission (10, 11). On the other hand, biopsy and resected specimens had a similar density of CD8^+^ cells with a high Ki67 expression. For some chronic diseases, such as HIV infection, cancer, and autoimmune diseases, the Ki67 expression pattern in T cells has been reported to evaluate antigen-specific T cell expansion (12, 13). Our results might reflect preservation of potential host immune activity against tumor cells during the sequential treatments. Furthermore, although CD20^+^ B cells were not found in the biopsy specimen, they were observed surrounded by CD8^+^ cells in the resected specimens. The existence of tumor-infiltrating B cells (TIL-Bs) had been reported as a factor predicting the efficacy of platinum-based chemotherapy in MIBC (14, 15). Moreover, TIL-Bs in the microenvironment of other solid tumors had been reported as a valid prognostic factor given that it provides costimulatory signals important for CD8^+^ T cell maintenance in tumor tissues (16). This might be one of the reasons for chemotherapy resistance in this case. In recent studies, infiltration of CD20^+^ B cells into tumor was associated with survival and immunotherapy response. The prognostic significance of tumor-infiltrating CD20^+^ B cell was generally concordant with that of CD3^+^ and/or CD8^+^ T cells, and the prognostic effect of T cells was generally stronger when tumor-infiltrating CD20^+^ B cells were also present (17–19). Our result suggested that pembrolizumab promoted activation of CD20^+^ B cell *via* stimulation of CD4^+^ cells or directed blocking PD-1 expression on CD20^+^ B cells, following increased CD20^+^ B cells, contributing to the pembrolizumab response, together with other immune cells, such as CD8^+^ cells.

We also investigated PD-L1 and PD-L2 expression in the biopsy specimen. Accordingly, PD-L1 was highly expressed on tumor cells, while PD-L2 was not expressed in the cancer microenvironment. Pembrolizumab had been found to promote significantly longer overall survival and better objective responses compared to chemotherapy in the patients who had a tumor PD-L1 combined positive score of 10% or more (5). In UTUC, PD-L1 positivity of tumor cells was an independent prognostic factor of favorable survival outcomes in organ confined disease cases, possibly indicating successful host immune activity and stimulation of PD-L1 expression by interferon-γ, which prevents progression beyond organ boundaries (20). Although the importance of PD-L2 expression in ureteral cancer has been unclear, previous studies have reported that tumor PD-L2 expression was associated with poor prognosis. These results supported the effectiveness of pembrolizumab, which promotes the activation of T and B cells and enhances the cytotoxic effect of lymphocytes. Currently, Wahlin et al. (15) reported that immune cell infiltration in both pre- and postsurgical samples of MIBC was an important prognostic factor, independent of neoadjuvant chemotherapy. To the best of our knowledge, this has been the first report to show a pathological complete response and changes in microenvironment following second-line neoadjuvant pembrolizumab in chemoresistant UTUC, with the complete response having been maintained for over a year. Currently, the patient has maintained a progression-free survival of 15 months with no evidence of disease during routine imaging examinations. Future plans will involve surveillance with clinical and routine imaging examinations until progression, projecting a possible reinduction of immunotherapy for achieving pathological complete response after chemotherapy followed by immunotherapy in neoadjuvant setting. We need to mention the limitation of this research being a single case report, which does not allow to draw a conclusion of pembrolizumab response. Additionally, pembrolizumab is not allowed for treatment in the neoadjuvant setting for urothelial carcinoma by health insurance in Japan. However, we believe that the information presented herein can contribute toward further understanding intratumoral immune cell behavior and expanding the potential therapeutic spectrum of immunotherapy including the neoadjuvant setting. Moreover, our data support the urgent need for more prospective trials that would help establish a better definition for immunological biomarkers to identify potential long-term responders.

This report showed that nephroureterectomy could be safely performed for UTUC after neoadjuvant therapy with pembrolizumab. Despite this being the first case report, the infiltration of CD8^+^ lymphocytes after pembrolizumab may be considered a good prognostic factor.

## Data Availability Statement

All datasets presented in this study are included in the article/Supplementary Material.

## Ethics Statement

The studies involving human participants were reviewed and approved by Iwate Medical University of Medicine Institutional Review Board (IRB 2019-083). Written informed consent to publish this case report was obtained from the patient prior to enrollment.

## Author Contributions

DI, SK, TN, and WO wrote the report. DI, HS, DT, RK, and WO cared for the patient. HS, DT, RK, and WO performed the surgery. KI, TT, and TS helped to establish the pathological diagnosis. All authors contributed to the article and approved the submitted version.

## Conflict of Interest

The authors declare that the research was conducted in the absence of any commercial or financial relationships that could be construed as a potential conflict of interest.
